# Abortion Reporting in the United States: An Assessment of Three National Fertility Surveys

**DOI:** 10.1007/s13524-020-00886-4

**Published:** 2020-05-26

**Authors:** Laura Lindberg, Kathryn Kost, Isaac Maddow-Zimet, Sheila Desai, Mia Zolna

**Affiliations:** Guttmacher Institute, 125 Maiden Lane, 7th Floor, New York, NY 10038 USA

**Keywords:** Abortion, Survey measurement, Fertility, Data quality

## Abstract

Despite its frequency, abortion remains a highly sensitive, stigmatized, and difficult-to-measure behavior. We present estimates of abortion underreporting for three of the most commonly used national fertility surveys in the United States: the National Survey of Family Growth, the National Longitudinal Survey of Youth 1997, and the National Longitudinal Study of Adolescent to Adult Health. Numbers of abortions reported in each survey were compared with external abortion counts obtained from a census of all U.S. abortion providers, with adjustments for comparable respondent ages and periods of each data source. We examined the influence of survey design factors, including survey mode, sampling frame, and length of recall, on abortion underreporting. We used Monte Carlo simulations to estimate potential measurement biases in relationships between abortion and other variables. Underreporting of abortion in the United States compromises the ability to study abortion—and, consequently, almost any pregnancy-related experience—using national fertility surveys.

**Electronic supplementary material:**

The online version of this article (10.1007/s13524-020-00886-4) contains supplementary material, which is available to authorized users.

## Introduction

Demographic research on fertility experiences relies on high-quality data from population surveys, particularly from respondents’ self-reports of births, miscarriages, and abortions.^1^ Yet prior studies have found that women severely underreport abortion in the National Survey of Family Growth (NSFG), a primary data source for study of American fertility experiences (Fu et al. 1998; Jones and Forrest 1992); for example, 47% of abortions were reported in the 2002 NSFG (Jones and Kost 2007). When respondents omit abortions from their pregnancy histories, the accuracy of these survey data is compromised. This limits not only research on abortion experiences but any research that requires data on all pregnancies, including research on pregnancy intentions, contraceptive failure, interpregnancy intervals, infertility, and any survey-based research on pregnancy outcomes for which pregnancies ending in abortion are a competing risk. Thus, abortion underreporting in population surveys has far-reaching implications for fertility-related research in demography and other fields.

There has been no rigorous examination of the quality of abortion reports in more recent U.S. fertility surveys. However, there are many reasons to hypothesize that previously documented patterns of abortion reporting may have changed given that multiple factors may be playing a role in respondents’ willingness to report their experiences in social surveys. The social and political climate surrounding abortion has become more hostile in recent years (Nash et al. 2016), which may have increased abortion-related stigma and women’s reluctance to disclose their experiences. If abortion underreporting is a response to stigma, then neither the level of stigma nor patterns of responses would necessarily be fixed over time. Widely declining survey response rates may indicate less trust in the survey experience (Brick et al. 2013) and impact people’s willingness to report sensitive behaviors, including abortion. Substantial and differential declines in abortion rates in the United States have changed the composition and size of the population of women with abortion experiences (Jones and Kavanaugh 2011), altering the population at risk of underreporting. These declines may also decrease women’s exposure to others who have had abortions, increasing the stigma of their experience (Cowan 2014). Abortion reporting may also be affected by the recent increases in medication abortion (Jones and Jerman 2017). All these factors may influence recent patterns of abortion reporting in the NSFG as well as other national surveys.

In this study, we present a comprehensive assessment of abortion underreporting in recent, widely used nationally representative U.S. surveys and its potential impact on measurement of fertility-related behaviors and outcomes. This work improves on prior analyses in several ways. We estimate levels and correlates of abortion reporting in the NSFG, National Longitudinal Survey of Youth 1997 (NLSY97), and National Longitudinal Study of Adolescent to Adult Health (Add Health) surveys, examining completeness of abortion reports by respondents’ characteristics. In addition, increased sample sizes in the redesigned NSFG continuous data collection permit more precise estimation than prior studies. By expanding the investigation to include the NLSY and Add Health, and comparing patterns of reporting between the three surveys, we illuminate a broader set of survey design issues that can be used to inform future data collection, including factors such as sampling and survey coverage, interview mode, and length of retrospective recall. Furthermore, although prior work has documented high levels of underreporting, it has offered limited guidance to researchers about how this may impact estimates when abortion data is used in analysis. This article presents the first demonstration of how underreporting may bias analyses that rely on these data, based on new Monte Carlo simulations. These findings are relevant not only for research on abortion, pregnancy, and fertility, but for any study that relies on respondents’ reports of stigmatized or otherwise sensitive experiences.

## Background

It is well documented that survey respondents may not fully report sensitive behaviors or experiences (Tourangeau and Yan 2007). There are varying types of sensitivity within the survey context, including threat to disclosure, intrusiveness, and social desirability (Tourangeau et al. 2000). Threats to disclosure refer to concrete negative consequences of reporting and are most relevant for illicit behaviors (i.e., drug use, criminal activity). For example, some women in the United States are not aware that abortion is legal (Jones and Kost 2018) and may fear legal consequences from disclosure. Sensitivity to intrusiveness is related to questions that are seen as an invasion of privacy, regardless of the socially desirable response. Finally, social desirability may prevent a respondent from revealing information about a behavior if the consequence is social disapproval, even if just from the interviewer. Abortion underreporting may reflect a deliberate effort to reduce any of these types of sensitivity. However, given the widespread political, social, and moral debates over abortion in the United States, we hypothesize that fear of social disapproval is most likely the reason women do not report abortions in surveys.

This social disapproval has been conceptualized as abortion stigma, the process of devaluing individuals based on their association with abortion (Cockrill et al. 2013; Kumar et al. 2009; Shellenberg et al. 2011). A national study in 2008 found that 66% of U.S. abortion patients perceived abortion stigma (Shellenberg and Tsui 2012). Studies have shown a positive link between women’s perception of abortion stigma and their desire for secrecy from others (Cowan 2014, 2017; Hanschmidt et al. 2016). This desire may influence how women respond to survey questions about abortion; that is, survey respondents may not report their abortion experiences in order to provide what they perceive as socially desirable responses and thus reduce their exposure to stigma (Astbury-Ward et al. 2012; Lindberg and Scott 2018; Tourangeau and Yan 2007).

### Previous Findings

Jones and Forrest (1992) pioneered a methodology to compare women’s reports of abortions in the 1976, 1982, and 1988 NSFG cycles to external abortion counts (Jones and Forrest 1992). They found that compared with external abortion counts, only 35% of abortions were reported across the surveys. They thus concluded that “neither the incidence of abortion nor the trend in the number of abortions can be inferred from the NSFG data” (Jones and Forrest 1992:117). Later analyses found that abortion reporting in the 1995 and 2002 NSFG remained substantially incomplete compared with external abortion counts (Fu et al. 1998; Jones and Kost 2007). Model-based estimates of abortion underreporting in the NSFG without an external validation sample also have found large reporting problems, but results are highly sensitive to alternate model specifications (Tennekoon 2017; Tierney 2017; Yan et al. 2012).

Incomplete reporting of abortion is not isolated to the NSFG; it has been documented in other national U.S. surveys, including the 1976 and 1979 National Surveys of Young Women (Jones and Forrest 1992; Zelnik and Kantner 1980) and the 1979 National Longitudinal Surveys of Work Experience of Youth (Jones and Forrest 1992). Other U.S. studies compared women’s survey reports with abortion counts obtained from Medicaid claims (Jagannathan 2001) or medical records (Udry et al. 1996), with similar findings of significantly incomplete reporting. Abortion underreporting also has been documented in France and Great Britain (Moreau et al. 2004; Scott et al. 2019) and in countries where abortion is illegal (Singh et al. 2010).

Women’s willingness to report an abortion may vary across individuals. Indirect evidence for this comes from research on abortion stigma finding that certain groups are more likely to perceive or internalize abortion stigma than others (Bommaraju et al. 2016; Cockrill et al. 2013; Frohwirth et al. 2018; Shellenberg and Tsui 2012). More directly, there is some evidence of variation in completeness of abortion reporting by women’s individual characteristics, including age, marital status, race/ethnicity, and religion, but patterns of differential underreporting have been inconsistent across studies and samples (for recent summaries of these patterns, see Tennekoon 2017; Tierney 2017).

Abortion reporting also might vary by the type or timing of a woman’s abortion. Medication abortion now represents more than one-third of all abortions and approximately 45% of abortions that occurred prior to nine weeks of gestation (Jones and Jerman 2017). Many women may choose medication abortion instead of a surgical procedure because they feel it is a more natural and private experience (Ho 2006; Kanstrup et al. 2018). And women may be less likely to report these abortions to protect their privacy, or they may not interpret the survey questions as referring to their experience. The 2002 NSFG showed some evidence of less complete reporting of abortions prior to nine weeks’ than at later gestations, but the relative incidence of medication abortions in the United States during the time covered by the survey was much lower than it is today (Jones et al. 2019), and differences in estimates by gestational age were not significant (Jones and Kost 2007). To date, little is known about how the increased use of medication abortion has affected abortion reporting in surveys. Additionally, regardless of the type of abortion, women may incorrectly report abortions at earlier gestational ages as miscarriages to reduce social disapproval (Lindberg and Scott 2018).

### Efforts to Improve Reporting

Most efforts to improve abortion reporting have focused on developing survey designs that seek to reduce sensitivity related to fear of social disapproval. For example, in 1984 and 1988, respectively, the NLSY and NSFG added a confidential self-administered paper-and-pencil questionnaire component that asked women to report their abortions (Mott 1985; U.S. Department of Health and Human Services (DHHS) 1990). In both, the self-administered question resulted in increased reporting of abortions compared with the interviewer-administered questions, but the numbers were still low compared with external counts (Jones and Forrest 1992; London and Williams 1990). Since 1995, the NSFG has supplemented the interviewer administered face-to-face (FTF) interview with audio computer-assisted self-interviewing (ACASI) for the most sensitive survey items, including abortion (Kelly et al. 1997; Lessler et al. 1994). As with the earlier self-administered paper-and-pencil questionnaire supplements, ACASI was developed to increase privacy and confidentiality (Gnambs and Kaspar 2015; Turner et al. 1998). Respondents listened to questions through earphones and entered their responses into a computer. Studies of the 1995 and 2002 NSFG found improved abortion reporting in the ACASI compared with the FTF interview (Fu et al. 1998; Jones and Kost 2007). The Add Health and NLSY97 surveys also used ACASI to supplement the interviewer-administered surveys, but the abortion questions were asked only on the ACASI.

Evidence from the United States and internationally suggests that other aspects of the survey and question design also influence abortion reporting. In the British National Survey of Sexual Attitudes and Lifestyles Survey, abortion reporting may have declined after a change from a direct question (ever had an abortion) to a more complicated pregnancy history (Scott et al. 2019). Similarly, in French data, a direct question on abortion resulted in increased reporting compared with a complete pregnancy history (Moreau et al. 2004). An analysis of the Demographic and Health Surveys (DHS) also found that longer and more complicated surveys resulted in less complete reporting of births, suggesting a reporting issue that is distinct from the sensitivity of the pregnancy outcome (Bradley 2015). Survey questions often ask respondents to focus on recent periods because of concerns that reporting quality deteriorates with more distant recall (Bankole and Westoff 1998; Koenig et al. 2006), but evidence of this pattern in abortion reporting is limited (Philipov et al. 2004).

### Comparing the NSFG, NLSY, and Add Health

In contrast to research on abortion reporting in the NSFG, limited information exists on the completeness of reporting in the NLSY97 or Add Health despite their use for studies of fertility and pregnancy experiences. Estimates of the completeness of abortion reporting in Add Health range widely from 35% (Tierney 2019) to 87% (Warren et al. 2010). We are not aware of any published analysis of the quality of NLSY reporting.

The different designs of the three surveys may influence respondents’ abortion reporting (Table 1). For example, compared with the cross-sectional data collection in the NSFG, the design of NLSY and Add Health may result in better reporting if respondents feel more invested in the longitudinal survey process; alternatively, they could have worse abortion reporting if women feel less anonymity (Gnambs and Kaspar 2015; Mensch and Kandel 1988). The length of recall for abortion also varies because of different survey and question designs. Additionally, NSFG asks about abortion in both the FTF and ACASI survey modes, whereas NLSY and Add Health rely only on the latter; if ACASI improves abortion reporting, we might expect both NLSY and Add Health to have better abortion reporting than the NSFG FTF interview.

**Table 1:** Descriptions of NSFG, NLSY97, and Add Health Surveys

	NSFG	NLSY97	Add Health
Study Design	Cross-sectional	Longitudinal	Longitudinal
Survey Sample	Nationally representative household survey of women aged 15–44	Nationally representative household survey of youth born between 1980–1984 and living in the United States at first 1997–1998 interview	Representative of students in grades 7–12 at time of 1994–1995 Wave 1 interview
Analysis Sample	Full sample	2013–2014 Round 16 respondents	Wave 4 respondents
Survey Round(s) Used in Analysis	2006–2010, 2011–2015	Rounds 12–16	Wave 4
Sampling	Multistage, stratified, clustered	Multistage, stratified, clustered	Multistage, stratified, school-based, clustered
Response Rate	78% women (2006–2010); 72% women (2011–2015)	82% women (Round 16)	80% (Wave 4)
IRB Approval	National Center for Health Statistics	The Ohio State University and NORC at the University of Chicago	University of North Carolina at Chapel Hill
Survey Mode^a^	FTF; ACASI	ACASI	ACASI
Length of Abortion Recall Period	Lifetime (FTF); last five years (ACASI)	Since last interview; recall may have been up to eight years	Lifetime
Date of Abortion	Month and year (FTF); unspecified within interval (ACASI)	Month and year	Month and year

^a^FTF = face-to-face interview; ACASI = audio computer-assisted self-interview.

The three survey systems also differ in sample composition and coverage (Table 1). The NSFG is a nationally representative household survey; the NLSY included only youth born between 1980 and 1984 and living in the United States at the time of the first 1997–1998 interview; and Add Health originally selected only students in grades 7–12. Thus, the three surveys differ in the extent to which women were excluded from the original sampling frame. This would influence the number of abortions reported compared with external counts for the full population.

This study evaluates the completeness of abortion reporting across the NSFG, NLSY, and Add Health to reveal the influence of survey design, including the use of ACASI, on reporting. To isolate the influence of the sensitivity of abortion on reporting, compared with other survey design factors (e.g., the sampling frame or nonresponse biases), we contrasted the patterns of completeness of abortion counts with population and birth counts—an approach recommended by recent research (Lindberg and Scott 2018). Additionally, we leveraged the increased sample size of the recent NSFG to provide a more robust examination of differential reporting by women’s characteristics and by timing of their abortion than was possible in prior analyses, including, for the first time, an examination of the influence of the length of retrospective recall on reporting. Finally, we investigated how differential underreporting of abortion may bias analyses.

## Data and Methods

### Data Sources

Data from three household surveys—the NSFG, NLSY, and Add Health—were used in this analysis. Table 1 and Table A1 of the online appendix provide details about the design of each survey and the specific abortion measurement items.

#### *National Survey of Family Growth*

The NSFG is a household-based, nationally representative survey of the noninstitutionalized civilian population of women and men aged 15–44 in the United States (Groves et al. 2009). To strengthen the reliability of estimates, we pooled data from women in the 2006–2010 (*n* = 12,279) and 2011–2015 (*n* = 11,300) surveys; these rounds asked identically worded abortion questions, and we found no differences in abortion reporting across these two periods. Female respondents were asked to report pregnancies and their outcomes first in the FTF interview and then again in ACASI. The FTF interview collected a lifetime pregnancy history,^2^ including the outcome (live birth, still birth, abortion, or miscarriage) and the date when the pregnancy ended. The ACASI asked for the number of live births, abortions, and miscarriages within the last five years, separately for each outcome.

#### *National Longitudinal Survey of Youth 1997*

The NLSY97 is a nationally representative, longitudinal survey of men and women born between January 1, 1980, and December 31, 1984 (Bureau of Labor Statistics 2019).

Respondents were interviewed first in 1997–1998, then every year through Round 15 (2011–2012), and then biennially through Round 17 (2015–2016). The NLSY User Services team confirmed a problem in the Round 17 “preload” information impacting how nonbirth outcomes were reported (Bureau of Labor Statistics 2018), so we included only the cohort of female respondents interviewed in the 2013–2014 Round 16 (*n* = 3,595).

In each survey round, women were asked via ACASI to report each pregnancy that ended since their last interview date, including the outcome (live birth, stillbirth, miscarriage, abortion) and end date. Birth counts for 2007–2011 were drawn from the Biological/Adopted Children Roster for Round 16 generated by NLSY survey staff, which provided birth dates of all biological children reported. No such data are available for abortions. Instead, we combined reports of abortions and their occurrence dates from women interviewed in Round 16 (2013–2014) as well as any abortions they may have reported during prior interviews to obtain all retrospective reports of abortions from these women during the five-year period from 2007 to 2011. Abortions for which we were not able to determine whether they occurred within this period were included only in sensitivity analyses.

#### *National Longitudinal Survey of Adolescent Health (Add Health)*

Add Health is a longitudinal, nationally representative survey of male and female students in grades 7–12 in the 1994–1995 school year (Harris 2013). Add Health used a multistage, stratified, school-based, cluster sampling design; adolescents who had dropped out or were otherwise not attending school at Wave 1 were not included. In Wave 1, 20,745 respondents were interviewed at home and followed up at three subsequent waves. We used the Wave 4 restricted data set, which included interviews with 7,870 of the original female respondents in 2008. In the Wave 4 interview, female respondents were asked via ACASI to provide a complete pregnancy history, including abortions, and the dates of each pregnancy. Although a full pregnancy history was also collected in Wave 3, and pregnancies prior to the first interview were asked about in Wave 1, high levels of missing dates for these pregnancies make it impossible to identify unique pregnancies across waves. This means that we could not combine reports across waves and had to rely solely on Wave 4 reports.

### External Counts of Abortions, Births, and Population

To assess completeness of abortion reporting in each survey, we compared respondents’ reports with the actual number of abortions that occurred in the United States for a matching period and corresponding population of women. We obtained these counts of abortions overall by year and by demographic subgroups from data collected by the Guttmacher Institute; we refer to these counts as being “external” to the survey.

Since 1976, the Guttmacher Institute has fielded the Abortion Provider Census (APC), a national census of all known abortion providers, to obtain numbers of abortions performed annually in the United States. Recent data collection efforts were designed to identify early medication abortions as well as surgical abortions (Jones and Jerman 2014). Although the APC aims to identify and contact all abortion providers, an estimated 4% of abortions are missed annually because some women obtain abortions from private practice physicians not identified in the census (Desai et al. 2018). Similarly, a small number of hospital-based abortions also are missed (Jones and Kost 2018). Still, the National Center for Health Statistics (NCHS) has historically used these data to calculate national pregnancy rates (Ventura et al. 2012) because the APC counts are considered the most complete data available. As such, they provide an external “gold standard” for this analysis; any undercount of abortions in the APC would underestimate the completeness of abortion reporting in fertility surveys.

To estimate the annual numbers of abortions in the United States in the period 1998–2014, we used data from rounds of the APC conducted in 2001–2002 (providing data for 1999 and 2000), 2006–2007 (data for 2004 and 2005), 2010–2011 (2007 and 2008 data), 2012–2013 (2010 and 2011 data), and 2015–2016 (2013 and 2014 data). Estimates for interim years were obtained from previously published interpolations (Jones and Jerman 2017).

The Guttmacher Institute’s periodic nationally representative Abortion Patient Survey (APS) collects information on the demographic characteristics of women obtaining abortions. We obtained annual distributions of these characteristics from 1998 to 2014 using linear interpolation of the cross-sectional distributions in the 1994, 2000/2001, 2008, and 2014 APSs (StataCorp 2017b:455) (see Table A2 of the online appendix for these distributions by year). These annual distributions were then applied to the total number of abortions from the APC for each year to obtain our external counts of annual numbers of abortions for multiple demographic groups.

Annual external counts of births were drawn from U.S. vital statistics (U.S. DHHS 2018a) and tabulations of counts of births by nativity status (National Center for Health Statistics 2018b). Annual external population counts were from the Census Bridged-Race Population Estimates (U.S. DHHS 2018b).

For each survey, external counts of abortions and births were adjusted for comparability and to take into account births and abortions that occurred to women not represented in the surveys’ sampling frames (see the online appendix). Most importantly, each survey includes a constrained age range of women, which itself varies each year in which the pregnancy could be reported. In addition, the NSFG interviews of women take place across multiple years, so the reporting period for abortions covers different time periods based on each woman’s interview year.

## Analytic Strategies

For each survey, we first compared estimated weighted population counts with external population counts to assess survey coverage and to identify the number of women missing from the sampling frame. Next, we assessed completeness of the weighted number of births reported in each survey compared with the external counts to help isolate the influence of the sensitivity of the pregnancy outcomes on reporting compared with other survey design factors. Finally, we calculated the proportion of external counts of abortions reported in each survey (using weighted numbers). For all estimates, we show 95% confidence intervals to account for survey sampling error. We assessed significance on the basis of nonoverlapping confidence intervals; this is a relatively conservative approach because it will fail to reject the null hypothesis (that the point estimates are equal) more frequently than formal significance testing (Schenker and Gentleman 2001). All analyses accounted for the complex survey design of each data set by applying sampling weights provided by each survey system and the *svy* commands in Stata 15.1 (StataCorp 2017a).

### NSFG

For the pooled 2006–2015 NSFG data, we obtained the annual weighted number of abortions and births in the five years preceding the January of the interview year, with separate estimates from the FTF and ACASI reports to compare with external counts.

We also calculated the proportions of abortions reported for gestational age and eight demographic characteristics that can be identified in both the NSFG and the external abortion data: age, race combined with Hispanic ethnicity, nativity,^3^ union status, religion, poverty status, current education level, and number of prior births. For births, we calculated proportions reported by age, and race combined with Hispanic ethnicity; these are the only comparable demographic variables available in both vital records data and the NSFG.

To determine whether abortion reporting deteriorates with longer or shorter recall periods, we compared abortion reporting in eight-, five- and three-year retrospective recall periods, using the lifetime pregnancy history in the FTF interview. We compared reporting from the five-year recall period in the FTF and ACASI interviews to examine variation in survey modes.

### NLSY97

We calculated the proportion of the external counts of abortions and births in 2007–2011 reported by women in the NLSY97 (see the online appendix). Because there were only 188 abortions (unweighted) reported in the period under study, we did not estimate differences by sociodemographic characteristics.

Observed discrepancies between the NSLY birth and abortion reports and the external counts may be driven by women missing from the original sampling frame. The NLSY97 included only women living in the United States at the time of screening in 1996; thus, women who immigrated to the United States after this year were not represented in the sample, although they did contribute to the external counts of abortions and births. The external data sources (APC, vital records, and census) did not have a measure of year of immigration, so we could not identify and exclude the experiences of women immigrating after 1996. Instead, we used measures of nativity to limit the NLSY97 and the external data sources to exclude foreign-born women, which allowed us to assess a more comparable second set of survey-based and external counts.^4^

### Add Health

Add Health was never a fully nationally representative survey: it was designed to be representative of students in grades 7–12, and the original sampling frame excluded out-of-school youth. Even if every woman in Add Health reported fully and accurately on her abortion experiences, these numbers would underestimate the national count of abortions because of differences in the populations covered by the two reporting systems.

To assess abortion reporting in Add Health, we adjusted the external abortion counts to exclude abortions occurring to women who would not have been in the original Add Health sampling frame. The original sample from students in grades 7–12 did not align directly with any particular age range in the external population counts. Our analytical sample excluded about 7% of respondents at the tails of the age distribution where fewer women in these ages would be eligible for inclusion in the sampling frame (because of the variation in the ages in which students enter 7th grade and exit 12th grade). We included only female respondents aged 26–31 at the time of the Wave 4 survey, resulting in an analytical sample of 7,357 female respondents aged 26–31 at the time of the Wave 4 survey. We contrasted reports of abortions and births among these respondents to adjusted external estimates in that same age range (see the online appendix). We did not estimate sociodemographic differences because there were only 529 abortions (unweighted), which would lead to unstable subgroup estimates.

We conducted a sensitivity analysis adjusting Add Health and the external data sources to be as comparable as possible in excluding those out of school. Our best approximation was to exclude from both the external counts and Add Health those births and abortions from women age 26–31 who had not graduated high school by the date of their reported pregnancy.

### Estimation of Bias from Abortion Underreporting

To illustrate the impact of abortion underreporting in studies using survey data, we followed an approach previously used to estimate bias introduced by misclassification of responses in binary regression (Neuhaus 1999). We conducted Monte Carlo simulations of the bias introduced by abortion underreporting in an analysis that uses reported abortions as an outcome. We model a hypothetical study that attempts to estimate the amount that some binary demographic characteristic (η) increases a woman’s likelihood of having had an abortion. In the absence of underreporting, this could be defined by the logistic regression model

$$ \log \left(\frac{p\left(Y=1\right)}{1-\kern0.28em p\left(Y=1\right)}\right)={\upbeta}_0+{\upbeta}_1\upeta, $$

where β_1_ is the covariate of interest, and *Y* is whether the respondent has had an abortion. However, we know that *Y* is measured with error; we observe only Y*, a measurement that has perfect specificity (all respondents reporting abortion have had an abortion) but poor sensitivity (high levels of underreporting).

To illustrate the bias induced by using Y*, we repeatedly sampled 10,000 women from a hypothetical population in which 30% had the characteristic η, and 40% of women, overall, would have reported an abortion if they had one. The bias induced by underreporting is the difference in the estimated relationship ${\hat{\upbeta}}_1$ (using Y* instead of *Y* as the response) and the true value of β_1_ (which we set to be held constant at 1; OR = 2.7). In each simulation, we systematically varied two factors that can influence the degree of bias: the extent of differential reporting (the amount η increases the likelihood of reporting) and the overall prevalence of abortion in the population. Each simulation scenario was run 50 times, and we calculated the average bias across the simulations.^5^

## Results

### Sample Distributions of Each Survey

Table 2 shows the unweighted sample size and weighted percent distribution of the analytic samples from the NSFG, NLSY, and Add Health surveys. Each NSFG survey sample had a similar demographic composition by age; over time, though, each has become more racially diverse, lower-income, less likely to be currently married, and more educated. The share of reproductive-age women reporting no religion also increased over time in the NSFG. By design, the NLSY97 and Add Health had narrower age distributions compared with the NSFG. Further, because the original samples of the NSLY and Add Health were drawn in 1996 and 1994–1995, respectively, more of the samples were non-Hispanic White than in the later NSFG surveys. Other differences in the demographic measures likely reflect the different age compositions of the NLSY97 and Add Health compared with the NSFG.

**Table 2:** Weighted distribution and unweighted sample sizes of analytical sample, by demographic characteristics and survey

	NSFG				NLSY97 Round 16		Add Health Wave 4	
	2006–2010		2011–2015					
	%	*N*	%	*N*	%	*N*	%	*N*
Total	100	12,279	100	11,300	100	3,595	100	7,357
Age at Interview								
<20	17	2,284	15	2,047				
20–24	17	2,098	17	1,913				
25–29	17	2,366	17	2,117	20	698	71	5,055
30–34	15	2,047	17	2,011	80	2,897	29	2,302
35 and older	34	3,484	33	3,212				
Race/Ethnicity								
White, non-Hispanic	62	6,301	58	5,285	67	1,667	68	3,996
Black, non-Hispanic	14	2,535	15	2,420	16	1,031	16	1,677
Other, non-Hispanic	7	720	8	743	5	118	4	453
Hispanic	17	2,723	20	2,852	12	772	13	1,222
Poverty Status^a^								
<100%	22	3,361	28	3,900	16	658	40	2,666
100% to 199%	23	2,994	21	2,525	18	629	26	1,866
200+%	54	5,924	51	4,875	66	1,949	33	2,449
Union Status at Time of Interview								
Married	41	3,971	38	3,410	49	1,568	56	4,006
Cohabiting	11	1,451	15	1,573	18	652		
Formerly married	9	1,260	8	1,110	7	256		
Never married	38	5,597	39	5,207	26	1,109	44	3,344
Level of Education								
<Grade 12	21	3,072	18	2,542	17	672	8	449
High school diploma or GED	27	3,323	24	2,916	38	1,469	14	999
Some college	28	3,339	30	3,348	9	322	44	3,297
College degree	24	2,545	28	2,494	36	1,118	33	2,611
Religion								
Protestant	48	5,756	47	5,518	–– ^b^	–– ^b^	54	3,963
Catholic	25	3,135	22	2,518	–– ^b^	–– ^b^	20	1,580
Other	9	1,037	8	849	–– ^b^	–– ^b^	8	616
None	18	2,351	22	2,415	–– ^b^	–– ^b^	18	1,175
Nativity Status								
Foreign-born	15	2,070	16	1,857	5	240	4	432
U.S.-born	85	10,206	84	9,440	95	3,355	96	6,925

^a^At the time of the interview. Measured as personal income in Add Health and as household income in NSFG and NLSY97.

^b^Not available.

### NSFG

#### *Population Size and Birth Counts*

The weighted population counts for the 2006–2015 NSFG were nearly identical to those of the external population counts, reflecting the NSFG’s use of poststratification weights to match population totals to census counts (Table 3). We estimated that the weighted number of births reported in the NSFG appears to be slightly larger than the external counts (107%, CI = 101–113),^6^ particularly among women aged 30 and older (110%, CI = 101–118) and non-Hispanic White women (110%, CI = 101–119). Thus, the sampling frame of the NSFG fully represented the number of women in the population and slightly overestimated the number of their births.

**Table 3:** Percentage of external counts for weighted population size of survey and for number of births reported in the five years prior to interview date, with 95% confidence intervals (CI) and unweighted survey counts: NSFG 2006–2015

	% Reported (NSFG / external counts × 100)	95% CI	Unweighted NSFG Count
Population Size	99	(93–104)	23,579
Births (Total)	107	(101–113)	8,948
Age at Birth			
<20	105	(92–117)	1,057
20–29	105	(98–112)	5,076
30 and older	110	(101–118)	2,815
Race/Ethnicity			
White, non-Hispanic	110	(101–119)	3,830
Black, non-Hispanic	107	(92–123)	2,069
Other, non-Hispanic	105	(74–136)	514
Hispanic	99	(84–115)	2,535
Survey Round			
2006–2010	107	(97–117)	4,728
2011–2015	106	(98–114)	4,220

#### *Abortion Counts*

In the 2006–2015 NSFG, 40% (CI = 36–44) of abortions in the prior five years were reported in the NSFG FTF interview compared with external counts (Table 4). We estimate similar proportions using a three-year (40%) or eight-year (39%) recall period, with overlapping confidence intervals. There also was no difference in the completeness of abortion reporting in the 2006–2010 and 2011–2015 survey rounds.

**Table 4:** Weighted number of abortions reported in the survey, external counts of abortions, percentage of external counts reported in the survey, 95% confidence intervals (CI), and unweighted number of abortions reported in the survey, by survey mode and length of recall: NSFG 2006–2015

	Weighted Number of NSFG Abortions	External Counts (adjusted)	% Reported (NSFG / external counts × 100)	95% CI	Unweighted Number of NSFG Abortions
Five-Year Recall FTF^a^	4,575,254	11,413,954	40	(36–44)	1,180
2006–2010	2,367,494	6,043,097	39	(33–45)	612
2011–2015	2,207,760	5,370,857	41	(35–47)	568
Three-Year Recall FTF^a^	2,663,486	6,731,802	40	(35–44)	705
Eight-Year Recall FTF^a^	7,143,890	18,499,516	39	(35–43)	1,787
Five-Year Recall ACASI^b^	8,272,507	11,413,954	72	(65–80)	1,976

^a^Face-to-face interview.

^b^Audio computer assisted self-interview.

Women reported nearly two times as many abortions in the last five years in the ACASI as the FTF interview. The ACASI abortion counts are 72% of the external counts. Because the ACASI asks only about the previous five years, other recall periods could not be examined.

### Reporting by Women’s Characteristics and Gestational Age

In the 2006–2015 FTF interviews, the completeness of abortion reporting generally was low for all demographic groups (Table 5). Women younger than age 20 at the time of their abortion and those in the highest income categories (at least 200% above the poverty status threshold) were the only demographic groups to report at least 50% of their abortions.

**Table 5:** Weighted number of induced abortions reported in the survey for five years prior and percentage reported relative to adjusted external counts along with 95% confidence intervals (CI), by survey mode and women’s characteristics: NSFG 2006–2015

	2006–2015: FTF^a^		2006–2015: ACASI^b^	
	% Reported (NSFG / external counts × 100)	95% CI	% Reported (NSFG / external counts × 100)	95% CI
Total	40	(36–44)	72	(65–80)
Age at Abortion				
<20	53	(43–62)	––^c^	––^c^
20–29	37	(32–42)	––^c^	––^c^
30 and older	40	(31–48)	––^c^	––^c^
Number of Births Prior to Abortion				
0	44	(38–51)	––^c^	––^c^
1 or more	37	(32–43)	––^c^	––^c^
Union Status at Abortion				
Married	34	(24–45)	––^c^	––^c^
Cohabiting	38	(31–45)	––^c^	––^c^
Formerly married	37	(27–48)	––^c^	––^c^
Never married	44	(39–50)	––^c^	––^c^
Weeks of Gestation				
<9	36	(31–41)	––^c^	––^c^
9–12	34	(28–41)	––^c^	––^c^
13+	78	(61–95)	––^c^	––^c^
Race/Ethnicity				
White, non-Hispanic	42	(35–50)	73	(61–86)
Black, non-Hispanic	41	(33–48)	71	(60–83)
Other, non-Hispanic	29	(16–41)	59	(29–89)
Hispanic	40	(32–48)	77	(61–94)
Poverty Status at Interview				
<100%	32	(26–38)	64	(54–75)
100% to 199%	34	(27–40)	63	(54–72)
200+%	55	(47–63)	90	(74–106)
Religion				
Protestant	43	(36–50)	85	(72–98)
Catholic	29	(22–36)	60	(47–74)
Other	39	(24–53)	68	(48–89)
None	47	(39–54)	69	(54–83)
Education at Interview				
<Grade 12	41	(32–51)	93	(72–113)
High school diploma or GED	46	(37–55)	79	(64–94)
Some college	38	(31–44)	62	(52–73)
College degree	33	(25–42)	62	(46–78)
Nativity Status^d^				
Foreign-born	26	(14–37)	83	(51–115)
U.S.-born	44	(38–50)	75	(65–84)
Survey Round				
2006–2010	39	(33–45)	69	(58–81)
2011–2015	41	(35–47)	76	(67–86)

^a^Face-to-face interview.

^b^Audio computer assisted self-interview.

^c^Not available.

^d^Nativity estimates reflect data from the 2011–2015 NSFG survey round.

Levels of abortion reporting vary substantially across subgroups of women in the FTF interviews. Subcategories by age, income, religion and nativity had nonoverlapping confidence intervals. For example, Catholic women reported only 29% (CI = 22–36) of their abortions compared with 47% (CI = 39–54) of women identifying with no religion. Foreign-born women had particularly poor reporting, with only 26% (CI = 14–37) of abortions reported among this group compared with 48% (CI = 38–50) among U.S.-born women.

We found no statistical differences in reporting for groups varying by parity, race/ethnicity, union status, or education; we also found no difference between the overall completeness of reporting in the 2006–2010 and 2011–2015 surveys.

There were large differences in reporting by gestational age in the FTF interview. Seventy-eight percent of abortions at 13 weeks’ or later gestation were reported, compared with 36% of abortions occurring at less than 9 weeks’ gestation and 34% at 9–12 weeks’ gestation.

Reporting of abortions in the ACASI was higher than in the FTF interview for virtually all the demographic groups identified (Table 5). With wider confidence intervals, there were no longer significant differences in reporting by any of the characteristics, although the direction of differences identified in the FTF measures remained.

### NLSY97

#### *Population Size, Birth, and Abortion Counts*

The NLSY97 2013 cumulative case sampling weights were designed to adjust the Round 16 respondents to represent the population of 9.4 million 12- to 16-year-olds as of December 31, 1996, adjusting for both the original sampling strategy and loss to follow-up. Thus, by design, the weighted NSLY97 sample in Round 16 (2013–2014) did not match the census counts for the later period: the national population has grown since 1996. Round 16 includes 89% of the population of women aged 29–33 nationally in 2013 (Table 6).

**Table 6:** Estimated number of women and their reported births and abortions in 2007–2011, and percentage reported relative to adjusted external counts: NLSY97, Round 16

	Weighted NLSY Count	External Counts (adjusted)	% Reported NLSY97 (external count × 100)	95% CI	Unweighted NLSY Count
All Women					
Population size^a,b^	9,438,553	10,663,010	89	(83–94)	3,595
Births^c^	4,977,553	5,787,668	86	(79–93)	1,938
Abortions^d^	437,223	1,470,682	30	(24–35)	188
Excluding Foreign-born					
Population size^a,b^	8,983,726	8,568,894	105	(98–111)	3,355
Births^c^	4,764,155	4,403,888	108	(99–118)	1,804
Abortions^d^	409,757	1,235,495	33	(27–39)	173

*Note:* The NLSY97 cohort is a longitudinal project that follows the lives of a sample of American youth born between 1980 and 1984; 8,984 respondents were aged 12–18 at first interview in 1997–1998.

^a^Population size was measured in NLSY97 Round 16 (2013–2014 interviews).

^b^External counts adjusted from the census and CPS.

^c^External counts adjusted from vital records.

^d^External counts adjusted from the Abortion Provider Census.

The weighted number of births reported for 2007–2011 represents 86% (CI = 79–93) of adjusted birth counts from vital records. This undercount closely parallels the population undercount, suggesting that the gap in birth counts was primarily accounted for by women missing from the sampling frame as opposed to underreporting of births by women who were interviewed. In contrast, women reported only 30% (CI = 24–35) of the abortions in the external counts for the same period (Table 6).^7^

To more directly examine the sensitivity of reporting to the exclusion of recent immigrant women in the NLSY97, we calculated a second set of estimates excluding all foreign-born women from both the NLSY97 and external counts. After this adjustment, the weighted number of women in the NLSY97 Round 16 was comparable to the population counts (105%, CI = 98–111), and 108% (CI = 99–118) of births were reported in the NLSY97 relative to external counts.^8^ However, despite this sampling frame adjustment, only 33% (CI = 27–39) of abortions were reported.

### Add Health

#### *Population Size, Birth, and Abortion Counts*

The number of women in Add Health Wave 4 aged 26–31 in 2008 was 82% of a nationally comparable population of women for the same year (Table 7). Women reported only 71% (CI = 63–80) of births in Add Health compared with vital records, and they reported 31% (CI = 25–37) of the external count of abortions. Thus, the abortion undercount was much larger than estimated for population or birth counts.

**Table 7:** Estimated number of women, and their reported births and abortions in 2003–2007, and percentage reported relative to adjusted external counts along with 95% confidence intervals (CI): Add Health, Wave 4

	Weighted Add Health Count	External Counts (adjusted)	% Reported (Add Health / external count × 100)	95% CI	Unweighted Add Health Count
All Women					
Population size^a,b^	10,029,020	12,183,021	82	(75–90)	7,357
Births^c^	4,848,152	6,794,737	71	(63–80)	3,555
Abortions^d^	615,780	1,969,832	31	(25–37)	529
Excluding Non–High School Graduates					
Population size^a,b^	8,476,026	10,294,653	82	(74–90)	6,392
Births^c^	3,972,409	5,524,597	72	(63–81)	3,004
Abortions^d^	481,352	1,733,861	28	(22–34)	443

^a^Population size was measured as of the Add Health Wave 4 interview.

^b^External counts adjusted from the census.

^c^External counts adjusted from vital records.

^d^External counts adjusted from the Abortion Provider Census.

Excluding women who are non–high school graduates from both Add Health and the external counts had little effect on our findings. After these women were excluded, the weighted number of women in the Add Health sample remained at 82% of the adjusted population counts; 72% (CI = 63–81) of the external counts of births were reported, and only 28% (CI = 22–34) of the abortions were reported.

### Estimation of Bias from Abortion Underreporting

**Fig. 1:**
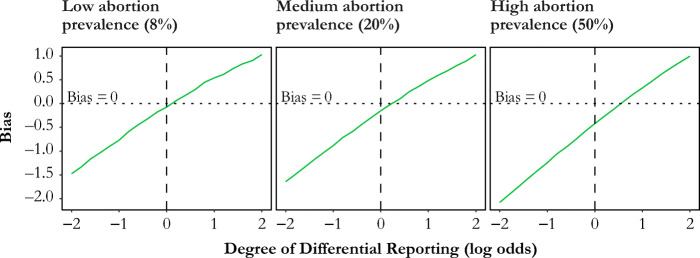
Bias in estimated relationship between an individual-level characteristic η and odds of abortion (expressed in log odds), according to overall abortion prevalence and degree of differential reporting by characteristic η, estimated using Monte Carlo simulations. True log odds (β_1_) are fixed at 1.

Figure 1 presents estimates of the bias induced by using underreported abortion data in logistic regression models in which having had an abortion is the outcome. Each panel corresponds to different levels of abortion prevalence: low (8% of women had abortions), medium (20% of women had abortions), and high (50% of women had abortions). The *y*-axis represents the average bias (the difference between the estimated relationship and the true relationship, expressed in log odds). The *x*-axis describes the degrees of differential reporting between groups, with more positive values indicating that women with characteristic η were more likely to report abortions, if they had any, than women without that characteristic.

Even relatively small amounts of differential reporting resulted in a substantial degree of bias in the estimated relationship between characteristic η and the likelihood of having an abortion. For example, in the low abortion prevalence setting, even a moderate negative relationship between η and reporting resulted in a severe underestimate of the true relationship; a moderate positive relationship resulted in a substantial overestimate. And, even in the absence of differential underreporting, the estimated relationship between characteristic η and the likelihood of having had an abortion was biased toward the null (the absence of a relationship), and this bias increased in settings in which overall abortion prevalence was higher.

The potential impact of these two factors—differential reporting and the overall prevalence of abortion—is difficult to predict, even under these simplified conditions. The two sources of bias can mitigate or exacerbate each other. For example, in our high abortion prevalence setting, there was downward bias even in the presence of a small positive relationship between η and underreporting. Comparatively, in the low abortion prevalence setting, this same relationship resulted in upward bias. In an analysis with real data, this problem would likely be compounded by the addition of other covariates, each of which may have their own (unknown) relationship with the likelihood of reporting. There may also be unobserved confounders that are associated with both the outcome and the likelihood of reporting.

## Discussion

Three prominent national surveys used widely for fertility-related research in the United States—the NSFG, NLSY97, and Add Health—have substantially incomplete reporting of abortions by women compared with external census-based counts of abortion. Overall, women reported only 30% to 40% of the external counts of abortions in FTF interviews in the NSFG and the ACASI in the NSLY and Add Health, with differentially incomplete reporting across social and demographic characteristics. There were no identified population groups reporting even close to the true number of abortions. Use of ACASI improved reporting in the NSFG up to nearly three-quarters of the external counts, but it still resulted in substantial abortion underreporting in the NLSY and Add Health.

Incomplete abortion counts in each survey appeared to be driven by underreporting of women *in* the survey, not those missing from the survey. Our analysis of population counts and birth counts in each survey as well as our sensitivity tests of compositional issues found little evidence that the undercount of abortions is attributable to the exclusion of women from the original sampling frame. The NSFG’s weighted population was roughly equivalent to the parallel external counts; its weighted birth counts in the last five years were slightly overestimated. This overestimate may be driven by misreporting of births that occurred prior to the past five years as having occurred during that period, particularly among older women who have had more time to experience a birth, and have more births to report than younger women. Incomplete reporting of abortion in these surveys appeared to be influenced by the stigma associated with abortion. Furthermore, our findings suggest that length of recall did not affect the quality of abortion reporting, which has implications for the design of new survey questions.

The increases in abortion reporting in the ACASI portion of the NSFG compared with the FTF interview is not surprising, given that ACASI is designed to provide privacy and improve reporting of sensitive behaviors. However, more reporting via ACASI does not necessarily mean less measurement error or more valid reports. In particular, some of the increased reporting of abortions via ACASI compared with the FTF interview may reflect women incorrectly shifting events into ACASI’s five-year reporting period and/or incorrectly reporting lifetime as opposed to recent pregnancies. In fact, the adoption of the five-year reporting window in the NSFG’s ACASI since 2006 may be inducing measurement error; earlier NSFG rounds, which asked women to report on lifetime number of abortions in both the FTF and ACASI interview, did not find as large an increase in reporting in the ACASI as estimated here. Thus, researchers should be wary of this additional source of measurement error in the ACASI reports of abortion in the recent rounds of the NSFG. Additionally, the ACASI does not ask follow-up questions or the date of the abortion. For example, the ACASI cannot provide information about age or marital status at the time of the abortion, nor how the timing of the abortion occurred relative to other pregnancies. Thus, the NSFG’s ACASI reports are likely insufficient for most research on abortion or pregnancy.

More generally, the ACASI methodology was not a universal fix to abortion reporting problems; both the NLSY and Add Health used ACASI to measure abortion with substantial underreporting. Instead, distinct NSFG design features may have facilitated higher ACASI reporting than in the NLSY and Add Health. For example, in the NSFG, each woman answered the ACASI questions after the FTF interview, where they had already been asked to report on abortion as part of a detailed pregnancy history. This may have primed respondents and provided a second chance to report abortion experiences. Furthermore, unlike NLSY and Add Health, the NSFG ACASI abortion question has an introduction designed to normalize the behavior and was a single item as opposed to a full pregnancy history. Future research should consider how abortion reporting is influenced by survey design factors separate from (or potentially interacting with) survey mode.

Despite increased restrictions on abortion in the United States, this analysis provides only a modest suggestion that underreporting of abortion has worsened. The completeness of abortion reporting in the 2006–2015 NSFG FTF interviews (40%, CI = 36–44) was smaller than in the 1995 NSFG (45%, CI not reported) and the 2002 NSFG (47%, CI = 40–55) but with overlapping confidence intervals (Fu et al. 1998; Jones and Kost 2007). We also did not find any indication that reporting of abortions prior to 9 weeks of gestation—more likely to be medication abortions—was less complete than what are likely surgical abortions at 9–12 weeks.

Certainly all survey data contain flaws and weaknesses, including measurement error. Still, most analyses depend on an assumption that measurement error is random and not systematic. Here, we identified measurement error that is nonrandom and large in magnitude. Not only are the majority of abortions missing in these data, but incomplete reporting of abortion occurs differentially. Our simulations found that with differential underreporting, estimated associations can be biased in ways that are unpredictable in both direction and magnitude. We observed differential reporting for some key population groups, but other differential reporting is also likely, including for characteristics that cannot be measured in this study. It is impossible to assume the implications of any unknown differential abortion underreporting because the direction of bias can be either toward or away from the null (Luan et al. 2005; Neuhaus 1999). In studies using pregnancy as an outcome, the bias may be smaller in magnitude (abortions account for less of the total) but is still both unpredictable and potentially substantial. Furthermore, analytic models including abortion or pregnancy as a covariate also risk bias because of unmeasured confounding (where propensity to report is the omitted covariate).

Researchers should also be concerned with measurement error from what women may choose to add to a survey in place of an omitted abortion. For example, women who do not report an abortion may adjust survey responses to report more consistent or correct contraceptive use than actually occurred. This has implications for how we understand patterns of contraceptive use, the likelihood of experiencing contraceptive failures, and other pregnancy-related outcomes from these surveys. Our analysis also identified high levels of missing data and coding issues in the pregnancy histories collected longitudinally in both Add Health and NLSY, which future research should consider.

We could not test directly whether some women misreported an abortion as a miscarriage; however, this likely did not occur with notable frequency. First, for some women, miscarriage also is a stigmatized pregnancy outcome (Bommaraju et al. 2016). Women are more likely to report a miscarriage via ACASI than the FTF NSFG interview, and thus FTF miscarriage counts are already underestimates (Jones and Kost 2007; Lindberg and Scott 2018). Second, although we might expect that abortions occurring at the earliest gestations would be more likely to be mislabeled as miscarriages, there is no evidence of more incomplete reporting of abortions before 9 weeks of gestation than at 9–12 weeks. Third, a comparison of pregnancy outcome dates in the 1995 NSFG (the last round to collect this information with ACASI) revealed relatively few abortions in the ACASI that were identified as miscarriages or ectopic pregnancies in the FTF interview.

Although sample weighting is designed to adjust for survey nonresponse generally, selective nonresponse of women with abortions could potentially influence the completeness of abortion reporting. However, prior evidence focusing on the association between abortion reporting and response propensities in the NSFG is conflicting and incomplete (Peytchev 2012; Peytchev et al. 2010). Additionally, we know little about abortion prevalence among women residing outside the households in the survey sampling frames, such as women who are homeless, incarcerated, or living in military quarters, although access to abortion is severely limited for these groups (Bronson and Sufrin 2019; Cronley et al. 2018; Grindlay et al. 2011).

Another limitation is that a small percentage of abortions obtained from private physicians and hospitals are known to be missing from the APC. This likely leads to a modest undercount of abortions in the APC; thus, abortion reporting in the three surveys is likely slightly *worse* than what is estimated here. Self-managed abortion likely occurs rarely and not enough to distort the current study’s results; different studies estimate that 2% to 5% of U.S. women report trying to end a pregnancy on their own, which is often unsuccessful (Grossman et al. 2010; Jerman et al. 2016; Jones 2011; Moseson et al. 2017). However, as access to clinic-based abortions faces mounting legal barriers, more individuals may self-manage their abortion at home (Aid Access 2019; Aiken et al. 2018). Because Internet and mail provision of abortion medication will not be counted in conventional censuses of abortion providers, it will become increasingly important to improve abortion measurement in individual-level population surveys. The findings from this study can help inform new question designs and wording to better measure abortion. Additionally, measurement approaches being tested in settings where abortion is illegal or highly stigmatized (including the best friend approach, anonymous third-party reporting, confidante reporting, the list method, and network scale-up methods) may become increasingly relevant in the changing U.S. context (Bell and Bishai 2019; Rossier 2010; Sedgh and Keogh 2019; Sully et al. 2019; Yeatman and Trinitapoli 2011).

This study’s findings support the conclusion that abortion data from these national surveys should not be used for substantive research. Survey documentation has explicitly discouraged researchers from using the abortion data since the 1995 NSFG (Centers for Disease Control and Prevention, National Center for Health Statistics 1997). The most recent guidance states, “As in previous surveys, the NSFG staff advises NSFG data users that, generally speaking, NSFG data on abortion should **not** be used for substantive research focused on the determinants or consequences of abortion” (National Center for Health Statistics 2018a:34, emphasis in original). The NLSY and Add Health do not provide this type of guidance, yet the extent of underreporting documented in this analysis suggests that it is also relevant. Moreover, the NSFG warning as written may be interpreted too narrowly. With documented misreporting of miscarriage (Lindberg and Scott 2018), in addition to the bias of abortion underreporting that impacts the measurement of abortions and pregnancies overall, we conclude that only the reports of births from these surveys can be used without concerns of incomplete and biased reporting. This places a severe limit on the breadth of research possible and brings to the forefront a significant survey measurement issue for which we need new approaches and investment in improvements to our survey designs. To accurately measure and understand U.S. fertility behaviors—including the role of abortion in women’s lives—we must improve existing methodologies, and develop new ones, for measuring abortion as well as other sensitive or stigmatized behaviors.

## Electronic Supplementary Material

ESM 1 (PDF 182 kb)

## Data Availability

All data sets from the National Survey of Family Growth used for this analysis are publicly available at https://www.cdc.gov/nchs/nsfg/index.htm; data sets from the National Longitudinal Survey of Youth 1997 are publicly available at https://www.nlsinfo.org/content/cohorts/NLSY97 and abortion counts from the Guttmacher Institute’s Abortion Provider Census can be found in the Guttmacher Data Center at https://data.guttmacher.org/states. Sources for birth and population counts for relevant years are cited in text and can be downloaded from CDC Wonder at https://wonder.cdc.gov/. This study used restricted data from the Add Health Survey, which is not publicly available but can be requested at https://data.cpc.unc.edu/. Additional data on mother’s nativity status that support the findings of this study are available from the National Association for Public Health Statistics and Information Systems, but restrictions apply to the availability of these data, which were used under license for the current study and thus are not publicly available.

## Footnotes

1 Many transgender men, gender-nonbinary, and gender-nonconforming people also need and have abortions. Unfortunately, we are unable to identify these populations in this study because the NSFG, NLSY, and Add Health are designed to measure self-reported sex as male or female. Furthermore, only survey respondents self-identifying as female are asked about their pregnancy history. We use the term “women” to describe respondents throughout this article because it best aligns with the majority populations in these national surveys. However, we acknowledge that these data limitations may exclude the experiences of some people obtaining an abortion or experiencing pregnancy.

2 In the FTF interview, a section on recent reproductive health care asked women whether they had an abortion in the 12 months preceding the interview. These responses could not be contrasted to a comparable external count but produced similar abortion counts to those reported in the pregnancy history for the prior 12 months.

3 Nativity data are available only for respondents interviewed in the 2011–2015 survey wave.

4 We adjusted population counts excluding foreign-born women of that age in the 2013 CPS. Birth counts excluding foreign-born women were obtained from NCHS. External abortion counts excluding foreign-born women were obtained by applying the proportion of abortions to non–foreign-born women from the APS survey to the total external count.

5 The complete code is available online: https://osf.io/z3nty/.

6 Separately estimated comparisons of birth counts for 2006–2010 and 2011–2015 each included 100 in their 95% CI.

7 The extent of abortion underreporting was not sensitive to the inclusion of nine additional abortions with ambiguous dates that may have occurred during the relevant time frame.

8 We estimate that immigrant women account for about 9% of births in the national counts.
