# Supplementary material for: Abortion Reporting in the United States: An Assessment of Three National Fertility Surveys
Source: Demography. 2020 May 26;57(3):899–925. doi: 10.1007/s13524-020-00886-4 (PMC7329789; doi:10.1007/s13524-020-00886-4)
Supplement: Supplementary file 1 — (PDF 182 kb) [file 13524_2020_886_MOESM1_ESM.pdf]

Online Appendix: Calculating external abortion counts

NSFG Adjustments

At time of interview, pregnancy histories are collected for each woman interviewed in the NSFG survey. For assessment of the completeness of abortion reporting in the NSFG, we examined all abortions reported as having occurred in the five years preceding the woman's year of interview. For example, if a woman was interviewed in 2007, we include all abortions she reported in each year from 2002 to 2006; abortions reported by women interviewed in 2006 contribute to the number of abortions for years 2001 to 2005, and those reported by women interviewed in 2008 are counted toward the total number reported in the NSFG survey for the years 2003 to 2007. These five year retrospective windows are applied to all women interviewed in each of the NSFG interview years, 2006-2015.

Two adjustments must be made *to the external counts of abortions* to match the abortions that could have been reported by NSFG respondents: an age adjustment and an interview year adjustment. We adjust the external counts of abortions using a series of assumptions about the distributions of respondents' actual age at abortion in each of the five years prior to, and not including, the year of the survey. In the method details below, these are referred to as "age adjustments". In addition, while the NSFG is designed to be a cross-sectional, nationally representative survey, data collection since 2006 is continuous and interviews from a single year are not representative of all women aged 15-44 (in addition, the distribution of women across interview years is unequal). We refer to adjustments made to account for the distribution of interviews across years within a "round" of the survey (e.g. the 2006-2010 NSFG and the 2011-2015 NSFG) as "interview year" adjustments.

Age adjustment

The NSFG respondents are a random sample of women ages 15-44 at time of interview. Respondents' retrospective reports of abortions—as well as abortions they may have had but did not report—refer to increasingly younger ages as the time between interview and the year of the abortion increases. As a consequence, when we rely on NSFG data, we are missing some abortions for women at older ages. For example, a woman who had an abortion at age 44 in 2003 would be age 47 in 2006, and so would not be included in the NSFG sampling frame. We thus

have to adjust the external count of abortions for prior years to remove abortions to women outside of the sampling frame. Failure to omit those abortions from the external count would result in an overestimation of abortion underreporting in the NSFG, affecting both the estimates of underreporting among the oldest women as well as the total among all women. Thus, for each of the five years prior to the interview year, we examine how old women who had abortions in the external counts would be at the time of the survey in order to include only those who match to NSFG respondents.

This correction is slightly more complicated than it first appears because of the interplay between a woman's date of birth, date of abortion and her date of interview. Most 44-year-old women who had abortions in 2005 (for example), would be 45 at the time they were screened for an interview in 2006 and would not be in the NSFG sampling frame for that interview year. Some proportion, however, would actually still be 44 at the time they were screened, and so would be included in the sampling frame (for example, if a woman with a June birthday had an abortion at age 44 in August of 2005, and was interviewed in May of 2006, no birthdays would have passed between her date of abortion and date of interview, and she would still be age 44 at the time of the interview). Another small proportion would be age 46 (for example, if a woman with a June birthday had an abortion at age 44 in May of 2005, and was interviewed in August of 2006). To determine, for each year, the proportion of women in the external data who are outside of the NSFG sampling frame, we would thus ideally need the exact dates the abortions occurred in each year and the birthdays of the women having those abortions. Unfortunately, we do not have external data to this level of granularity, so we make several simplifying assumptions. For the most part, NSFG interviews occurred throughout the year (with the exception of the first and last years of survey interviews; more on this below). We assume that birthdates and dates of abortion are also equally distributed throughout each year and throughout each month.[1] Finally, we assume that for abortions that occurred in the same month as a woman's birthday, ½ occurred to women at the younger age (before her birthday) and ½ occurred at the older age (after her birthday). These assumptions allowed us to calculate adjustment factors to apply to the number of abortions to women at each individual age in the external counts.

---

[1] There is some evidence there may be seasonality of abortions such that they are not evenly distributed throughout the year, but we do not expect that any skewing in the distribution would have a significant impact on counts of abortions by age.

Once we adjust external abortion counts by age to match the ages of women reporting in the NSFG, we sum the adjusted counts to obtain a new, adjusted total number of annual abortions appropriate for matching to the fertility survey.

Interview year adjustment

A final adjustment is needed because the NSFG surveys from 2006 on are designed as a nationally-representative, cross-sectional survey, but all interviews are not conducted in a single year or even a short range of years (e.g. 2 or 3). For example, the 2006-2010 survey responses, when weighted, refer to a representative population during the five year time period. Only 13% of the total 2006-2010 sample were interviewed in 2006. Thus, we should not expect these women to be able to represent all abortions that would have occurred during a five year time period prior to 2006. We adjust the external accounts by a factor that corresponds to the sum of the proportion of interview years reporting for that year. For example, all interview years can report abortions for 2005, but only those interviewed in 2006 and 2007 can report abortions for 2002; therefore we adjust the external, comparison count of abortions for 2002 to be equal to 39% of the total for that year (13% + 26%). The table below shows the proportion of respondents interviewed in each year by “round” of the NSFG (2006-10 and 2011-15).

2006-2010 NSFG

Year of Interview	Proportion of sample interviewed (weighted)
2006	13
2007	26
2008	24
2009	24
2010	13

2011-2015 NSFG

Year of Interview	Proportion of sample interviewed (weighted)
2011	6
2012	24
2013	26
2014	25
2015	18

In addition, we account for the timing of the interviews in the first and last years of the survey. Interviewing for the 2006-2010 NSFG was conducted from June 2006 through June 2010,[2] and interviews for the 2011-2015 NSFG were conducted from September 2011 through September

[2] From 2006-2010 User’s Guide: “Interviewing for the release of the 2006-2010 NSFG was conducted from June 2006 through June 2010.” 2006-08 NSFG interviewing started in June 2006 (39 obs), but mostly got underway in July (202 obs). Similarly, it tapered off in June 2010 (86 obs; down from 233 in May). Calculations for adjustment factors assume interviewing begin in July 2006 and continued through June 2010.

2015.[3] The years with partial interviewing (2006 and 2015) contribute additional information to our age adjustment calculations and are accounted for in our algorithm for the age adjustment factors. An identical method is used to adjust external counts of births.

NLSY adjustments

The NLSY includes in its sampling frame all women born in the years 1980-1984, for whom we collect abortion reports for the years 2007-2011. For each of the years of the reporting period, we use the corresponding external data from the Abortion Provider Census, and can estimate, using data from the Abortion Patient Survey, the proportion of women having abortions in that year who were born in 1980-1984. However, because the Abortion Patient Survey collects integer age at time of abortion, as opposed to birth year, there is some ambiguity about whether women having abortions at the youngest and oldest ages corresponding to the NSLY ages could have been part of the NLSY sampling frame. For example, a woman having an abortion at age 27 in 2007 could have been born in either 1980 (in which case they would be part of the NLSY sampling frame) or in 1979 (in which case they would not be), depending, as in the case of the NSFG, on the date of the abortion in relation to their date of birth. We assume in these cases that there is a 50% probability that the woman is within the NLSY sampling frame, and adjust the external counts accordingly. An identical method is used to adjust external counts of births.

Add Health adjustments

We limited our sample of Add Health respondents to women aged 26-31 in Wave 4 (interviewed in 2008), and collect abortion reports from those women for the years 2003-2007. For each year of abortion, we use data from the Abortion Patient Survey to adjust our external count to only include women that were likely aged 26-31 in 2008 (see table below). An identical method is used for births, limiting to specific age ranges using vital statistics.

Year of abortion	Age range
2007	25-30
2006	24-29

[3] From 2011-2015 User's Guide: "Fieldwork for the 2011-2015 NSFG was conducted from September 2011 through September 2013." Few interviews were conducted in September 2011 (n=8). Calculations for adjustment factors assume interviewing began in October of 2011 and continued through September of 2015.

2005	23-28
2004	22-27
2003	21-26

Because the Add Health sample is restricted to high school students, and the sampling frame is not all U.S. women of a particular age, we did not make assumptions about the exact ages of the external sample (e.g. by accounting for month of abortion and month of birth). However, we did attempt to adjust for educational attainment since some women who did not complete high school would not have been included in the Add Health sample. To estimate external counts of abortions for women with at least 12 years of education, we estimated from the 2000 and 2008 Abortion Patient Survey the proportion of women in each of the age ranges above with a high school education or higher. We then used linear interpolation to estimate these proportions in the intervening years, and applied the relevant proportion to the abortion total for that age range in each year. For birth totals, we use a similar method, estimating the proportion of women in a given age range with more than 12 years of education using birth certificate data from each year.[4]

[4] Because of changes to the birth certificate reporting form over time, these data were only available for some states; for the purposes of this estimate, we assumed that the proportions for those states were identical to the proportions for the states without data.

Table A1 Interview questions from each survey

	NSFG	NLSY97	Add Health
Interview question	FTF[a] Q: In which of the ways shown on this Card did the pregnancy end?<br><br>1 Miscarriage<br>2 Stillbirth<br>3 Abortion<br>4 Ectopic or Tubal Pregnancy<br>5 Live birth by Cesarean section<br>6 Live birth by vaginal delivery<br><br>ACASI[b] Q: Between January (year of interview-5) and December (year of interview-1), how many pregnancies did you have that ended in abortion?	Q: Since the last interview, how many of your pregnancies were not live births, that is, they ended in a stillbirth, a miscarriage or an abortion?<br>*Now loop over all of the reported non-live births one at a time until all are accounted for:*<br>Q: In which month did you have the first stillbirth, miscarriage or abortion, that is, when did the pregnancy end?<br>Q: In which year did you have the first stillbirth, miscarriage or abortion, that is, when did the pregnancy end?<br>....<br>Q: What was the outcome of the pregnancy? Was it a:<br><br>1 Stillbirth<br>2 Miscarriage<br>3 Abortion	Q: How did this pregnancy end?<br><br>1 Pregnancy has not yet ended<br>2 Live birth<br>3 Single, stillbirth<br>4 Miscarriage<br>5 Multiple, involving both a live birth and another outcome<br>6 Multiple, no live birth<br>7 Abortion

[a] Face-to-face interview.
[b] Audio computer assisted self-interview.

Table A2 Percentage distribution of abortion patients by demographic characteristics and year: Abortion Patient Survey 1994, 2000, 2008, 2014[a]

	Year																				
	**'94**	'95	'96	'97	'98	'99	**'00**	'01	'02	'03	'04	'05	'06	'07	**'08**	'09	'10	'11	'12	'13	**'14**
Age at Abortion																					
<20	**21.5**	21.1	20.7	20.3	19.9	19.5	**19.1**	18.9	18.7	18.5	18.3	18.1	17.9	17.7	**17.5**	16.6	15.6	14.7	13.8	12.8	**11.9**
20-24	**32.8**	32.8	32.9	32.9	33.0	33.0	**33.0**	33.1	33.2	33.2	33.3	33.4	33.4	33.5	**33.6**	33.6	33.6	33.6	33.6	33.6	**33.6**
25-29	**21.4**	21.7	22.0	22.3	22.6	22.8	**23.1**	23.3	23.4	23.5	23.7	23.8	24.0	24.1	**24.3**	24.6	25.0	25.4	25.7	26.1	**26.5**
30-34	**14.4**	14.3	14.1	14.0	13.8	13.7	**13.5**	13.5	13.6	13.6	13.6	13.6	13.6	13.6	**13.6**	14.0	14.4	14.8	15.1	15.5	**15.9**
35 and older	**9.8**	10.1	10.3	10.5	10.7	11.0	**11.2**	11.2	11.2	11.1	11.1	11.1	11.1	11.1	**11.1**	11.2	11.4	11.6	11.8	12.0	**12.1**
Number of Births Prior to Abortion																					
0	**45.4**	44.3	43.3	42.2	41.2	40.1	**39.1**	39.1	39.1	39.2	39.2	39.2	39.3	39.3	**39.3**	39.5	39.8	40.0	40.2	40.4	**40.7**
1 or more	**54.6**	55.7	56.7	57.8	58.8	59.9	**60.9**	60.9	60.9	60.8	60.8	60.8	60.7	60.7	**60.7**	60.5	60.2	60.0	59.8	59.6	**59.3**
Weeks of Gestation																					
<9	**51.9**	53.4	54.8	56.3	57.7	59.2	**60.6**	60.8	61.0	61.1	61.3	61.5	61.7	61.8	**62.0**	62.3	62.7	63.0	63.3	63.6	**63.9**
9-12	**34.5**	33.8	33.0	32.2	31.4	30.6	**29.8**	29.6	29.4	29.2	29.1	28.9	28.7	28.5	**28.3**	27.9	27.5	27.1	26.7	26.3	**25.9**
13+	**13.5**	12.9	12.2	11.6	10.9	10.3	**9.6**	9.6	9.6	9.6	9.6	9.6	9.6	9.6	**9.6**	9.7	9.8	9.9	10.0	10.1	**10.2**
Race/Ethnicity																					
White, non-Hispanic	**45.8**	45.0	44.2	43.4	42.6	41.7	**40.9**	40.4	39.8	39.3	38.7	38.2	37.7	37.1	**36.6**	36.9	37.3	37.7	38.0	38.4	**38.7**
Black, non-Hispanic	**28.7**	29.2	29.7	30.2	30.7	31.2	**31.7**	31.4	31.1	30.8	30.5	30.2	29.9	29.6	**29.3**	29.0	28.7	28.4	28.2	27.9	**27.6**
Other, non-Hispanic	**5.3**	5.6	6.0	6.3	6.6	7.0	**7.3**	7.6	7.8	8.1	8.4	8.7	8.9	9.2	**9.5**	9.4	9.3	9.2	9.1	9.0	**8.8**
Hispanic	**20.2**	20.2	20.1	20.1	20.1	20.1	**20.1**	20.6	21.2	21.8	22.4	23.0	23.5	24.1	**24.7**	24.7	24.7	24.8	24.8	24.8	**24.8**
Poverty Status																					
<100%	**25.4**	25.6	25.8	26.0	26.2	26.4	**26.6**	28.5	30.5	32.4	34.4	36.3	38.2	40.2	**42.1**	43.3	44.5	45.7	46.9	48.1	**49.4**
100-199%	**24.4**	25.5	26.5	27.6	28.7	29.7	**30.8**	30.3	29.7	29.2	28.7	28.1	27.6	27.1	**26.6**	26.4	26.3	26.1	26.0	25.8	**25.7**
200+%	**50.2**	48.9	47.7	46.4	45.1	43.9	**42.6**	41.2	39.8	38.4	37.0	35.6	34.1	32.7	**31.3**	30.3	29.2	28.1	27.1	26.0	**25.0**
Union Status at Time of Abortion																					
Married	**18.4**	18.2	18.0	17.7	17.5	17.3	**17.0**	16.8	16.5	16.2	15.9	15.6	15.3	15.0	**14.8**	14.7	14.6	14.5	14.5	14.4	**14.3**
Cohabiting	**16.7**	18.1	19.6	21.1	22.5	24.0	**25.4**	25.9	26.4	26.8	27.3	27.8	28.2	28.7	**29.2**	29.5	29.8	30.1	30.4	30.7	**31.0**
Formerly married	**13.8**	13.3	12.8	12.3	11.9	11.4	**10.9**	10.9	10.9	10.9	11.0	11.0	11.0	11.0	**11.0**	10.6	10.3	9.9	9.5	9.2	**8.8**
Never married	**51.1**	50.4	49.6	48.9	48.1	47.4	**46.6**	46.4	46.2	46.0	45.8	45.6	45.4	45.3	**45.1**	45.2	45.3	45.5	45.6	45.8	**45.9**
Level of Education																					
<Grade 12	**21.0**	20.8	20.5	20.3	20.1	19.8	**19.6**	19.4	19.2	18.9	18.7	18.5	18.3	18.0	**17.8**	16.9	16.0	15.1	14.2	13.3	**12.4**
High school diploma or GEI	**30.4**	30.5	30.5	30.6	30.7	30.8	**30.9**	30.7	30.5	30.3	30.1	30.0	29.8	29.6	**29.4**	29.4	29.3	29.3	29.2	29.2	**29.1**
Some college	**34.9**	35.1	35.3	35.6	35.8	36.0	**36.3**	36.2	36.2	36.2	36.2	36.2	36.2	36.2	**36.2**	36.6	37.1	37.6	38.1	38.6	**39.1**
College degree	**13.7**	13.7	13.6	13.5	13.4	13.4	**13.3**	13.7	14.1	14.5	15.0	15.4	15.8	16.2	**16.6**	17.1	17.6	18.0	18.5	19.0	**19.4**
Religion																					
Protestant	**37.6**	38.5	39.4	40.3	41.2	42.1	**43.0**	42.3	41.6	40.9	40.2	39.5	38.7	38.0	**37.3**	36.1	34.9	33.7	32.5	31.3	**30.1**
Catholic	**30.5**	30.0	29.4	28.8	28.3	27.7	**27.1**	27.2	27.4	27.5	27.6	27.7	27.8	27.9	**28.0**	27.3	26.6	25.9	25.2	24.4	**23.7**
Other	**7.5**	7.5	7.5	7.4	7.4	7.4	**7.4**	7.3	7.3	7.2	7.2	7.1	7.1	7.0	**7.0**	7.2	7.4	7.6	7.8	8.0	**8.2**
None	**24.4**	24.1	23.8	23.5	23.1	22.8	**22.5**	23.2	23.8	24.4	25.1	25.7	26.4	27.0	**27.6**	29.4	31.1	32.8	34.6	36.3	**38.0**
Nativity Status																					
Foreign-born	—[b]	—[b]	—[b]	—[b]	—[b]	—[b]	—[b]	—[b]	—[b]	—[b]	—[b]	—[b]	15.9	15.9	**16.0**	16.0	16.0	16.0	16.1	16.1	**16.1**
U.S.-born	—[b]	—[b]	—[b]	—[b]	—[b]	—[b]	—[b]	—[b]	—[b]	—[b]	—[b]	—[b]	84.1	84.1	**84.0**	84.0	84.0	84.0	83.9	83.9	**83.9**

[a] Distributions for 1994, 2000, 2008, and 2014 (in bold) are based off data from the corresponding Abortion Patient Survey; linear interpolation methods are used to calculate distributions for intervening years.

[b] Note available.
